# Supplementary material for: Prototyping in Polymethylpentene to Enable Oxygen-Permeable On-a-Chip Cell Culture and Organ-on-a-Chip Devices Suitable for Microscopy
Source: Micromachines (Basel). 2024 Jul 10;15(7):898. doi: 10.3390/mi15070898 (PMC11278790; doi:10.3390/mi15070898)
Supplement: Supplementary file 1 [file micromachines-15-00898-s001.zip › micromachines-3034679-supplementary.pdf]

SUPPLEMENTARY INFORMATION TO

Prototyping in Polymethylpentene to Enable Oxygen Permeable on-a-Chip Cell Culture and Organ-on-a-Chip Devices Suitable for Microscopy

S1 Photographs of the prototyped PMP films

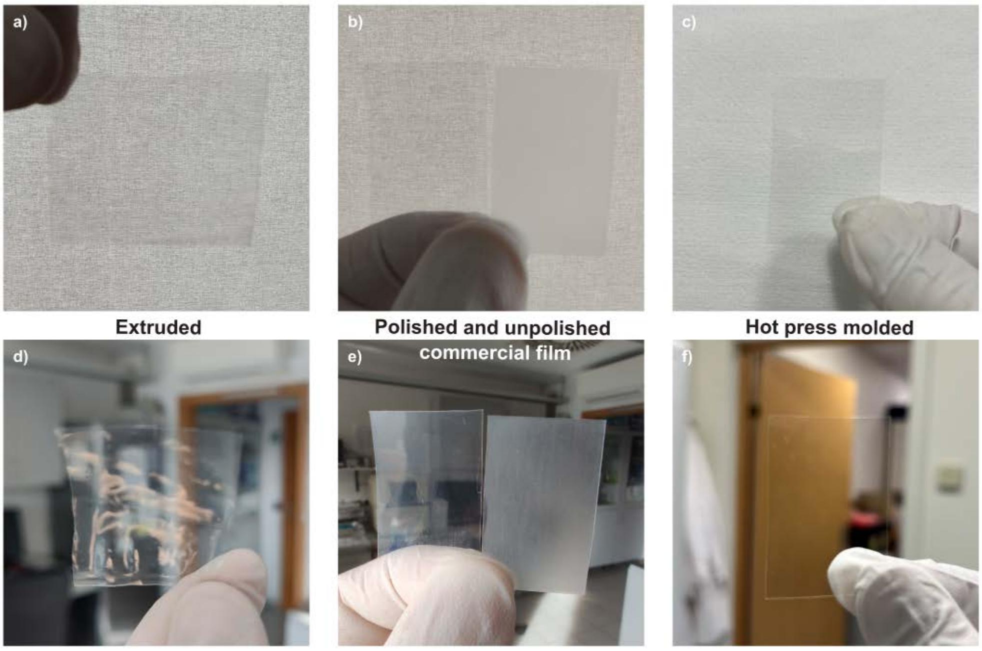

Figure S1. Photographs of (a,d) the extruded film, (b, e) the polished (left) and unpolished (right) commercial film and (c, f) the hot press molded film.

S2 Comparison of cell adherence, proliferation and viability on hot press molded PMP with or without plasma treatment prior to poly-L-lysine coating

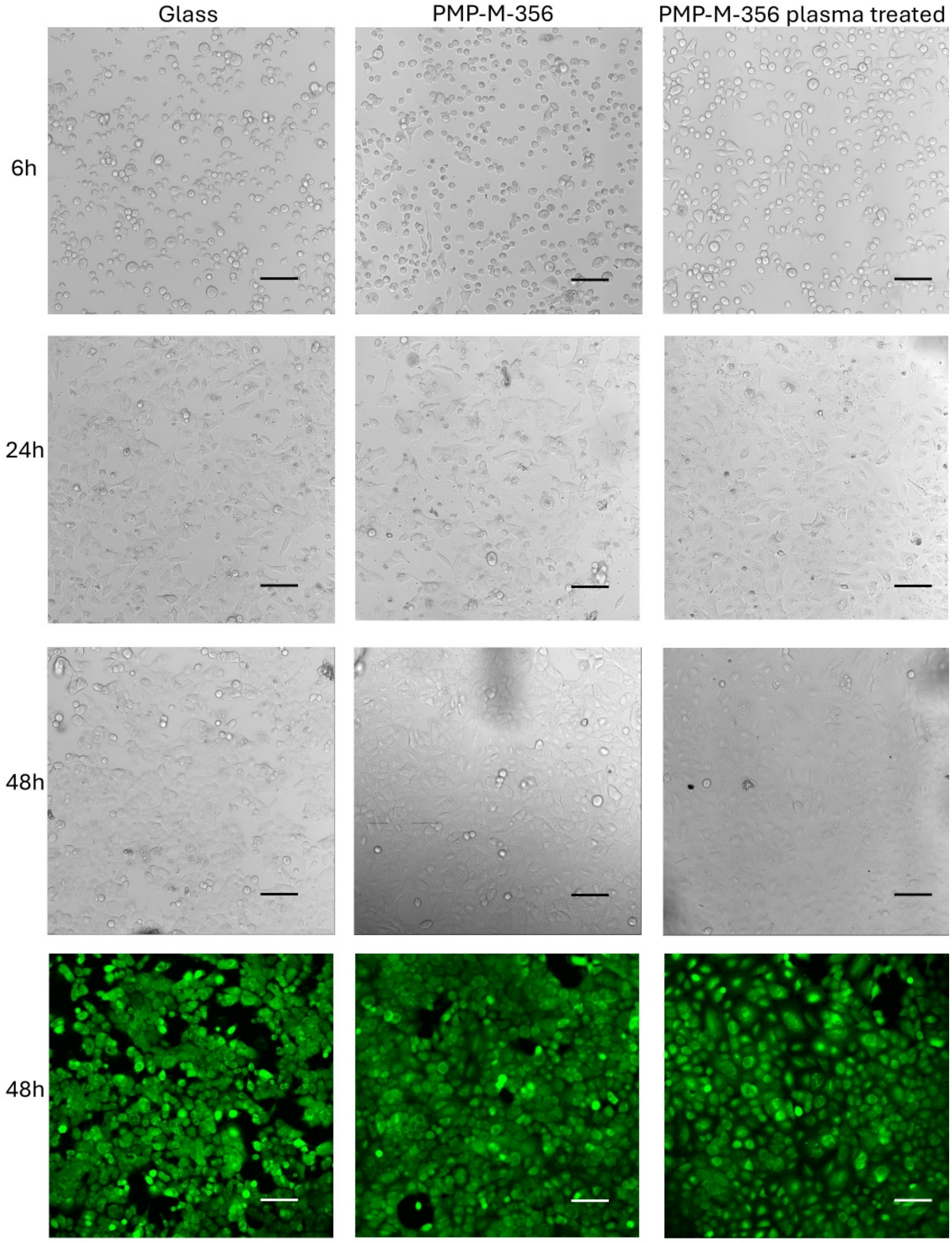

Figure S2. Microscopy images showing cell adherence, proliferation and viability of A549 cells cultured in Non-sensor devices with either glass coverslips, PMP-M-356 films or plasma treated PMP-M-356 films as layer 1 in the device, i.e., the cell-adhering layer. All materials were coated with poly-L-lysine prior to cell seeding. Transmission light images at 6, 24 and 48 hours follow the cell growth, while the green fluorescence at 48 hours display calcein-AM staining, where green indicates living cells. Scale bar: 100 μm.
